# Impact of the Healthcare System, Macro Indicator, General Mandatory Quarantine, and Mask Obligation on COVID-19 Cases and Death in Six Latin American Countries: An Interrupted Time Series Study

**DOI:** 10.3389/fpubh.2020.607832

**Published:** 2020-12-16

**Authors:** Adriana Poppe

**Affiliations:** Faculty of Management, Economics and Social Science, University of Cologne, Cologne, Germany

**Keywords:** COVID-19, Latin America, coping strategies, macro indicators, ITSA = interrupted time-series analysis, country comparison, mandatory quarantine, mask obligation

## Abstract

**Background:** Different coping strategies have been implemented by various governments worldwide to address the emerging health crisis of COVID-19. While most developed countries count on supporting healthcare and social systems, developing countries face additional challenges due to low macro indicators. The implementation of measurements such as quarantine are shown to be successful to flatten the curve of infection and death. In this context, it is important to test whether those measurements have an impact on the distribution of cases of COVID-19 in developing countries that face additional challenges such as lack of social security due to informal employment. A country comparison for Colombia, Costa Rica, Peru, Ecuador, Mexico, and Chile has therefore been conducted.

**Method:** The healthcare systems and macro indicator as well as the distribution of death due to COVID-19 per thousand inhabitants are compared descriptively. Using Multiple Interrupted Time Series Analysis with synthetic control units the impact of the General Mandatory Quarantine in Colombia, Peru, and Ecuador as well as the impact of Mask Obligation in public in Colombia and Chile have been tested.

**Results:** No clear impact of the poverty headcount ratio at the national poverty line and urban population on the percentage of death within the confirmed cases has been found. The out-of-pocked spending within health expenditure as a barrier in access to healthcare can be considered as a determinant of death within the confirmed cases of COVID-19. The implementation of a general mandatory quarantine did not show a curve-flattening effect in Ecuador and Peru but did so in Colombia. The implementation of Mask obligation in public spaced showed positive impact on the distribution of confirmed case in both countries tested.

**Conclusion:** The implementation of a general mandatory quarantine does not guarantee the curve-flattening effect. Various macro indicators should therefore always be considered while analyzing the effect of policies.

## Introduction

A new coronavirus (SARS-COV-2/COVID-19) emerged on December 12, 2019 in Wuhan, China (1). In the following months, the disease spread around the world. The World Health Organization (WHO) declared the coronavirus outbreak a Global Public Health Emergency on January 20, 2020. On March 11, the WHO determined that COVID-19 can be characterized as a pandemic (2). Due to the high number of cases and the rapid spread, healthcare systems are facing the most serious global pandemic crisis in a century [(3), p. 1].

The lack of knowledge about remedies and vaccinations are a problem straining the stability of healthcare systems (4, 5). The need to implement policies to reduce the incidence of infection is in danger of overloading hospital capacities and healthcare systems. The healthcare systems in Latin American Countries (LAC) have been shaped by the history of the worst income inequalities worldwide [(6), p. 1230]. Universal access to healthcare is included as a basic right in the constitution of each country (7, 8). However, the availability and the access to healthcare, even in countries with universal coverage, are unequal [(3), p. 3]. Accordingly, nearly 30% of the people in the lowest income quintile forgo care because affordability in OECD countries (3). With the increasing spread of SARS-COV-2 viruses, the demands on these unevenly distributed healthcare systems are growing. The critical task of healthcare systems is to protect the health of all citizen, especially in times of pandemics such as COVID-19 [(8), p. 9].

Many healthcare systems in LAC are characterized by fragmentation because providing a clear typology for health coverage is a difficult endeavor [(9), p. 15]. In some countries, such as Mexico, coexisting models and overlapping coverage makes it difficult to define the percentage of population with healthcare coverage (7, 10, 11). In this mode, being affiliated or contributing to a health system does not necessarily guarantee effective access or the quality of services received [(10), p. 38]. The generally weak and fragmented health systems are even more strained by the COVID-19 pandemic, as they have already been hit by Zika and Chikungunya outbreaks [(10, 12), p. 38]. A syndemic^1^ of measles, dengue, and COVID-19, among others, makes it all the more important for countries in the region to keep COVID-19 cases low (12). For example, in Ecuador, 82.57% of the confirmed COVID-19 cases and 84% of dengue cases are present in the coast and the city of Guayaquil (13). Efforts to stop the spread of the virus could be undermined by gaps in access to health services and the quality received [(10), p. 38]. The lack in the availability of intensive care units and specific diagnostic tests has been a concern regarding the upcoming health crisis (12). A baseline scenario during the outbreak of an healthcare crisis is imbalance between supply and demand for medical resources, which may grow rapidly in many countries [(14), p. 1]. To face the demand surge from COVID-19, health workforces, such as doctors and nurses, are key indicators of a timely and effective response [(8), p. 10]. The number of beds to cope with the increasing demand for hospital service due to the spread of the virus is indicative of how prepared the healthcare systems are (8).

With the aim to measure the general access to healthcare, Out-of-Pocket (OOP)^2^ may be considered, as it highlights barriers to access. On average, 34% of the total health spending in LAC are OOP [(8), p. 9]. On the OECD average, the OOP expenditures are above 21% (8). It can be said that a higher level of OOP spending indicates weaker healthcare Systems in the LAC with lower levels of health service coverage and an overall worse baseline scenario to confront health crisis (8). The basic characteristics of the healthcare systems are displayed in Table 1.

**Table 1:** Indicator of the Healthcare Systems (latest year available).

	**Hospital beds × 1,000 inhabitants**	**Doctors × 1,000 inhabitants**	**Nurses × 1,000 inhabitants**	**Out-of-pocket (OOP) share of health spending (%)**
Chile	2.1	2.5	2.7	34
Colombia	1.7	2.2	1.3	16
Costa Rica	1.1	3.1	3.4	22
Ecuador	1.5	2.0	2.5	39
Mexico	1.4	2.4	3.9	41
Peru	1.6	1.3	2.4	28

*OECD (8)*.

The spread of the virus, and with this, the likelihood of the healthcare system to collapse, is influenced by various macro indicators. Firstly, higher population density may increase the chances of human interaction [(15), p. 117]. Due to higher population density, the human interactions may increase, which favors the spread of viruses (15). In the past, it has been shown that densely populated urban areas have been more likely to be affected by epidemics of respiratory diseases, such as in the influenza pandemic of 1918–1919 (16). Secondly, age and underlying health conditions have been shown to be indicators determining the likelihood of infection, critical conditions, and consequently passing away due to the infection (17). Dowd et al. (18) showed a higher fatality among countries with a higher share of older citizen compared to younger societies. The likelihood of entering a critical condition thus increases with age, which leads to a higher demand for hospital care units within the healthcare system. It can thus by hypothesized that the hospitals are facing a higher demand with an increase in the share of older populations, which can increase the likelihood for a collapse of the healthcare system.

In LAC, a high degree of informality and inequality make the situation potentially more catastrophic compared to other parts in the world [(8), p. 11]. A lack of social protection likely results in the need to continue to work to make a living, which limits the capability to follow social distancing measures (8). Moreover, the possibility of working from home, overcrowded conditions, and lack of access to water and sanitation restricts the capability of individuals to cope with health emergencies such as COVID-19 [(4, 19, 20), p. 5]. Supporting the inequalities based on the access to clean water, Brojas (20) found a higher probability of having a positive COVID-19 test result for people living in poor neighborhoods, in neighborhoods where large numbers of people reside together within the same household, and in neighborhoods with a large black or immigrant population, like in New York [The United States (U.S.)]. Based on this, the macro indicators of the countries under study are displayed in Table 2.

**Table 2:** Marco indicator (latest date available).

	**GDP per capita in $ (2018)^**a**^**	**Population density (2018)^**b**^**	**Poverty headcount ration at national poverty lines (%)^**c**^**	**Population aged 65+ (%)^**d**^**	**Urban population (%)^**e**^**	**Sanitation (%)^**d**^**	**Access to drinking water (%)^**d**^**
**Chile**	14,670	25.189	8.6 (2017)	11.530	84.8%	100	100
**Colombia**	6,180	44.749	27.0 (2018)	8.478	80.4%	90	97
**Costa Rica**	11,520	97.913	21.0 (2019)	9.550	80%	98	100
**Ecuador**	6,110	68.789	25.0 (2019)	7.157	63%	88	94
**Mexico**	9,180	64.915	41.9 (2018)	7.224	83.9%	91	99
**Peru**	6,470	24.992	20.5 (2018)	8.088	79.1%	74	91

a *Worldbank (21)*.

b *Worldbank (22)*.

c *Worldbank (23)*.

d *OECD (8)*.

e *Worldometer.info (24)*.

In order to narrow the gap between medical need and available supply of treatments, public health measures known to reduce viral spread, such as social distancing and hand hygiene, may be implemented [(14, 25), p. 3]. During the implementation of measures against the spread of the virus, policy makers must draw on knowledge from previous pandemics and epidemics. A useful reference in the evaluation of possible policies aiming to flatter the curve of SARS-COV-2 is SARS-COV (SARS) (25). To control person-to-person transmission, measures such as isolation, quarantine, social distancing, and community containment were implemented in the main affected countries of China, Taiwan, Hong Kong, Singapore, and Canada in order to lower the transmission of the virus (25). Patients suspected of having SARS were isolated in either their homes, a hospital, or in government-designated places (e.g., hotels) until SARS could be ruled out (25). Individual interactions were reduced, responsibility to self-identify the disease and social distance were encouraged, and cancellation of public gatherings and implementation of community quarantine were introduced (25).

Researchers have already conducted studies testing the efficiency of various measures against the spread of COVID-19. Figueiredo et al. (26) have shown that the social distancing measures in two Chines provinces were effective in reducing incidences and mortality rates of COVID-19 (26). It has been shown that the effectiveness of lockdown policies declines with GDP per capita, population density and surface area and it increases with health expenditure and proportion of physicians in population (15).

Most of the Latin American Countries (LAC) remembered the lessons learned during SARS-COV and the influenza pandemic of 2009 (12). However, the strategies aiming to lower the infected and death by COVID-19 vary. A range of non-pharmaceutical Interventions (NPI) have been implemented, including closure of schools, mandatory healthcare coverage, mandatory quarantine, and aiming to increasingly reduce the population contact rates and slow the transmission of the virus. The present study focuses on six Latin American countries, Chile, Colombia, Costa Rica, Ecuador, Peru, and Mexico. The selection of the countries was made based on the availability of data on health systems in the case of Colombia, Chile, and Mexico as OECD countries. In addition, COVID-19 infection and death rates have been considered to allow the formation of synthetic cohorts. Furthermore, the countries were selected according to the implemented policies, so that countries with different coping strategies are included. Table 3 shows the main policies aiming to reduce the spread of the virus implemented the countries under study. All countries included in the study had implemented at least six policies by May 17 (27).

**Table 3:** Number of actions (regarding health) implemented by the countries (state of May 17th).

	**Chile**	**Colombia**	**Costa Rica**	**Ecuador**	**Mexico**	**Peru**
Health emergency	1	3	2	1	1	1
Mandatory coverage	0	1	1	1	0	0
Mandatory quarantine for foreign travelers, confirmed or suspected cases	1	1	1	1	0	1
Mandatory general quarantine	0	5	0	3	1^a^	1
Type of policy on testing (universal, reduced to certain groups, etc.)	2	1	3	1	1	3
Free test coverage expansions	1	2	2	1	1	0
Hospitals	3	2	6	0	1	0
Face masks in public transport/closed public spaces	1	1	0	0	0	0
Other	0	1	3	2	1	0
Total	9	17	18	10	6	6

*CEPAL and United Nations (27)*.

a *Not mandatory yet*.

The aim of this study is to analyze whether and to what extent the implementation of a general mandatory quarantine and mask obligation in public spaces affect the distribution of COVID-19 cases. In addition, the impact of resources in the healthcare systems and several macro indicators of the death due to COVID-19 will be described. For this purpose, a data set was assembled from various data sources. The individual sources are Our World in Data based on the European Center for Disease Prevention and Control, the websites of the governments of the countries included, the World Bank, WorldOMeter, and OECD. By now, various studies have been conducted to test the effect of implemented policies on the curve of cases and death due to COVID-19. However, most are conducted for industrial countries such as U.S.A., China or Spain (20, 26, 28). This study therefore aims to close the research gap by conducting a country comparison of the influence of macro indicators in six different LAC in order to provide deeper knowledge about the spread of COVID-19.

## Data and Methods

### Data and Variables

The data for the analyses were obtained from various sources. For this reason, the data origin is described together with the description of the variables so that it is possible to determine which data source is relevant for each variable.

Data of confirmed COVID-19 case per million inhabitants and the fatality per million inhabitants were obtained by Our World in Data (29). The platform collects data published by the European Center for Disease Prevention and Control (ECDC) and makes it available for free (29).

For the purpose of measuring the effect of the implemented policies, the last date of observation is set on May 24, as this was the beginning of the relaxing of the restrictions. Data starting February 29 until May 24 are included in the dataset. However, the starting point for each country is set to the first confirmed case and until 77 days after for each country. Since SARS-CoV-2 has an average incubation period of 5.1 days, with 97.5% of cases progressing to COVID-19 at around 11.5 days, it is assumed that the cases diagnosed in the first days after the implemented policies were infected before the implementation (26, 30). For this reason, a delayed effect of the implemented policies must be assumed.

Missing values in the data of confirmed cases and death of COVID-19 were found in the following cases and dates: Costa Rica on March 8 and Ecuador on March 7 and March 8. In the case of both cases, the missing values were found in the first week after the confirmation of the first COVID-19 value in the country. Since the number of reported new cases has been below 5 in both cases before and directly after the event, it has been assumed that no new cases were reported on the missing dates. The missing dates were therefore imported with the value 0 for new cases and death.

Data of the implemented policies are taken from the websites of the governments of the countries studied (31–35). With the aim to prevent errors in the data collection process, the collected data is double checked with OECDs report “COVID-19 in Latin America and the Caribbean: An overview of government responses to the crisis” (2020). The implemented policies are coded according to the date of implementation after the first confirmed case as dummy variables (0/1).

The macro indicators in the study are collected from the “World Bank World Development Indicators,” “WorldOMeter,” and “OECD” (see Table 1) (23, 24). All platforms make macro indicators from different countries available for use free of charge.

### Methods

#### Interrupted Time Series Analysis

All analyses will be done using STATA 15.1 and Microsoft Excel 365. The analyses are organized as follows. First, descriptive analysis of the impact of macro indicator on the distribution of cases and death is provided. Second, an Interrupted Time Series Analysis (ITSA) is conducted to examine whether the implementation of a certain policy has taken a decreasing effect on the distribution of the cases per million inhabitants.

ITSA is a quasi-experimental design with which longitudinal effect of interventions can be modeled though regressions. It is run by the STATA command *itsa* (36). Due to the data structure, statistical analysis used for ITSA must account for auto correlated data [(36) f]. In order to do so, an Ordinary Least Squares (OLS) regression model designed for autocorrelation using Newey-West estimators is employed, which controls for autocorrelation and heteroscedasticity in the error terms [(37), p. 639].

In order to specify the lags of the serial correlation in the data, the STATA command *actest* is used (38). It performs a Cumby-Huizinga general test for autocorrelation in time series data with the null hypothesis that serial correlation exists in the time series, but it dies out at a known finite lag (*q* > 0) (38). The lag in which the series correlation dies out will be included into the ITSA model in order to control for it.

In this study, the outcome variable in both cases are the confirmed cases per million inhabitants. The time elapsed since the start of the study is measured in days. The ITSA assumes the following form (36, 39):

$$\begin{array}{l} Y_{t}=\beta_{0}+\beta_{1}T_{t}+\beta_{2}X_{t}+\beta_{3}X_{t}T_{t}+\epsilon_{t} \end{array}$$

*Y*_*t*_ indicates the outcome variable measured at each time point t. β_0_ represents the starting level (intercept) of the outcome variable. β_1_ is the prior intervention trend, β_2_ represents the immediately occurring change in the level of the outcome variable after the introduction of the intervention, β_3_ is the treatment effect over time, which is the difference between pre-intervention and post-intervention slops of the outcome, and ϵ_*t*_ represents the random error term. Due to the incubation time of COVID-19, the analysis will focus on β_3_ rather than β_2_. A single-group ITSA is designed without a comparable control group; it rather projects the pre-intervention trend into the treatment period, which serves as the counterfactual [(36), p. 482].

Considering the multiple-group ITSA, the main assumption tested is that the exogenous policy shift affects all the groups [(36), p. 484]. The change in the outcome variable is therefore presumed to be the same for both the control and the treatment group (36). The regression equation is expanded by four additional terms (β_4_ to β_7_) [(36), p. 483]. A dummy variable to denote the cohort assignment (treatment or control) Z is introduced.

$$\begin{array}{l} Y_{t}=\beta_{0}+\beta_{1}T_{t}+\beta_{2}X_{t}+\beta_{3}X_{t}T_{t}+\beta_{4}Z+\beta_{5}ZT_{t}+\beta_{6}ZX_{t}\\ +\beta_{7}ZX_{t}T_{t}+\epsilon_{t} \end{array}$$

In the case of the multiple-group ITSA β_0_ to β_3_ represent the values of the control group and β_4_ to β_7_ represent the values of the treatment group. Going into detail, β_4_ represents the differences between treatments and controls prior to the intervention in the intercept of the outcome variable. β_5_ represents the prior intervention difference in the slope of the outcome variable. β_6_ represents the difference between treatment and control immediately following the introduction of the intervention and β_7_ represents the difference between treatment and control in the slope after initiation of the intervention comparing with pre-intervention.

#### Synthetic Control Unit

A synthetic control unit is a comparison unit as a linear combination of the untreated units with coefficients that sum to one [(40), p. 7] in order to test against the counterfactual. It is estimated by a weighted average of the untreated units that closely match the treated unit over the pre-treatment period [(41), p. 843]. The estimation is done using the STATA package synth (40–42). In order to test whether the synthetic cohort serves as a valid counterfactual, some outcomes in the pre-treatment period are excluded from the list of predictors to check whether the synthetic control matches well with the treated unit in these periods [(41, 43), p. 838].

## Results

The dataset includes the confirmed cases and deaths in Chile from March 4 to May 22, in Colombia from March 7 to May 22, Costa Rica from March 7 to May 22, in Ecuador from March 1 to May 19, Mexico from February 29 to May 15, and Peru from March 7 to May 22.

### Macro Indicator and Healthcare System

The analyses of the impact of the healthcare systems and macro indicator on the distribution of death and cases is based on the descriptive statistics. The dependent variable is the provenance of deaths as a proportion of the number of confirmed cases per million inhabitants per day. The values show that in Ecuador 14.50% of the confirmed cases died on day 77. In Mexico 10.50% died, in Colombia 3.60%, in Peru 2.90%, in Costa Rica 1.10%, and in Chile 1%.

Firstly, it is assumed that a higher poverty headcount ratio at national poverty lines and a lower percentage of the population living in urban areas leads to an increase in the percentage of death within the confirmed COVID-19 cases.

Table 4 indicates that Mexico has the highest poverty headcount ratio at the national level (41.9%) and the second highest percentage of death within the confirmed cases (10.5%). Chile shows the lowest poverty headcount ratio in terms of the national poverty lines (8.6%) and the lowest percentage of death within the confirmed cases (1%). These findings are in line with the assumption that a higher poverty headcount ratio at national level leads to a higher percentage of death within the confirmed cases. However, Ecuador displays a lower poverty ratio (25%) compared to Colombia (27%) but a higher percentage of death within the confirmed cases (14.5% Ecuador; 3.5% Colombia).

**Table 4:** Percentage of death within the confirmed cases and macro indicators.

	**Chile**	**Colombia**	**Costa Rica**	**Ecuador**	**Mexico**	**Peru**
% of death within the confirmed cases	1.00	3.60	1.10	14.50	10.50	2.90
Poverty headcount ration at national poverty lines (%)	8.6	27.0	21.0	25.0	41.9	20.5
Urban Population (%)	84.8	80.4	80	63	83.9	79.1

Regarding the influence of the percentage of the population living in urban areas, the lowest amount is shown by Ecuador (63%) and the highest amount is shown by Chile (84.8%). In addition, Ecuador shows the highest percentage of death within the confirmed cases (14.5%), and Chile shows the lowest percentage of death within the confirmed cases (1%). The findings reveal no clear trend for the poverty headcount ratio at the national poverty lines nor for the percentage of the observable urban population.

Now we turn to the assumption that higher resources in the healthcare system of a country lead to a lower percentage of death within the closed cases. The reported resources in the following are always to be interpreted as resources per 1,000 inhabitants. The results in Table 5 indicate no visible direction of the influence of the resources in the healthcare systems on the percentage of death within the confirmed cases regarding the resources in total (summed up). However, no trend within the distribution of the resources is visible in the sense that none of the countries have reported a low/high number of beds, doctors, and nurses. Chile, as the country with the lowest percentage of death within the confirmed cases, has the highest amount of hospital beds (2.1) and a high number of doctors (2.5). However, Costa Rica, as the country with the second lowest percentage of death, has the lowest number of hospital beds (1.1) and the highest number of doctors (3.1). The number of doctors in Mexico (2.4) is nearly as high as in Chile (2.5), but the percentage of death within the confirmed cases is the second highest (10.5%). A possible explanation could be an uneven distribution of hospital beds within countries. It is conceivable that the cases clustered occur in certain regions that may not have enough beds available. It is therefore not the total number of beds in the country that is relevant but the number of beds in the regions concerned.

**Table 5:** Percentage of death within the confirmed cases and healthcare systems.

	**Chile**	**Colombia**	**Costa Rica**	**Ecuador**	**Mexico**	**Peru**
% of death within the confirmed cases	1.00	3.60	1.10	14.50	10.50	2.90
Resources (per 1,000 inhabitants)	2	2	3	2	3	2
Hospital beds (per 1,000 inhabitants)	2.1	1.7	1.1	1.5	1.4	1.6
Doctors (per 1,000 inhabitants)	2.5	2.2	3.1	2	2.4	1.3
Nurses (per 1,000 inhabitants)	2.7	1.3	3.4	1.5	3.9	2.4
Out-of-pocket (OOP) share of health spending (%)	34	16	22	39	41	28

The results in Table 5 indicate that the countries with the highest share of OOP spending in the health spending show the highest percentage of death within the confirmed cases (Ecuador 14.5%; Mexico 10.5%). However, the share of OOP spending is higher in Mexico compared to Ecuador, and the percentage of death is lower. The share of OOP spending of Peru and Chile are higher compared to Colombia even though they report a lower percentage of death. The same is true for Costa Rica. The country with the lowest share of OOP spending does not report the lowest percentage of death among the cases.

### Implemented Policies

To investigate whether the implemented policies have an influence on the distribution of the confirmed cases and death due to COVID-19, ITSA was employed. The first model includes the implementation of the policy at the time of entry into force. In the second model, a delay of 14 days is included as second interruption time point in order to control for the expected time lack due to the incubation time. Due to the expected time delay between implementation and change in confirmed cases, the intercept will not be discussed in the analysis. Finally, multiple ITSA under the use of the synthetic control unit, following the same method as the single ITSA, are conducted.

To estimate the effect of the implementation of the general mandatory quarantine in Colombia, Peru and Ecuador directly after the implementation and 14 days later various ITSA models have been estimated. Table 6 presents the parameter estimates. This analysis examines the hypothesis that the implementation of a general mandatory quarantine has a decreasing effect on the distribution of confirmed cases.

**Table 6:** Results single ITSA–General Mandatory Quarantine.

	**Colombia**				**Ecuador**				**Peru**			
	**Implementation of**		**+** **14 days**		**Implementation of)**		**+** **14 days**		**Implementation of**		**+** **14 days**	
	**the Intervention**		**delay**		**the Intervention**		**delay**		**the Intervention**		**delay**	
	**(Model 1a)**		**(Model 1b)**		**(Model 2a)**		**(Model 2b)**		**(Model 3a)**		**(Model 3b)**	
	**β-Coefficient**	**Std. error**	**β-Coefficient**	**Std. error**	**β-Coefficient**	**Std. error**	**β-Coefficient**	**Std. error**	**β-Coefficient**	**Std. error**	**β-Coefficient**	**Std. error**
Pre-intervention intercept	−0.942**	0.345	−0.942**	0.350	0.201***	0.022	0.201***	0.022	−0.139**	0.045	−0.139**	0.046
Pre-intervention slope	0.271***	0.042	0.271***	0.042	0.096***	0.004	0.096***	0.004	0.160***	0.011	0.160***	0.011
Immediately post (day of implementation)	−48.488*	22.63	0.763	0.677	−374.069**	149.866	−13.942***	3.556	−681.412*	307.459	−0.432	0.236
Difference between pre- and post-Intervention slopes (day of implementation)	5.244***	0.754	1.548***	0.096	34.131***	3.578	7.245***	0.485	45.727***	8.231	1.323***	0.034
Immediately post (14 days delay)			−36.925	18.838		−233.389**	3.062			−588.751*	225.507	
Difference between pre- and post-Intervention slopes (14 days delay)			5.106**	0.796		34.720***	3.062			59.412***	7.368	

Significance levels: *p < 0.05, **p < 0.01, ***p < 0.001.

Focusing on the slope, ITSA identified significant interruptions in both time points for Colombia (Model 1B). The starting level of cases per million inhabitants is −0.942 (*p* ≤ 0.00) with an increasing slope in comparison with the pre-intervention period of 1.548 (*p* ≤ 0.00) at the first interruption point. The second interruption point has shown in comparison with the pre-intervention period an increasing slope of 5.106 (*p* ≤ 0.01). In other words, the distribution increased after both interruption time points in comparison with the pre-intervention period.

The estimates of the difference between pre-intervention and post-intervention at both time points (the actual implementation day and 14 days later) show that the difference of the slope is statistically significant for all three countries. However, the estimated coefficients of Colombia 14 days after the implementation (ß = 5.106) are smaller compared to Ecuador (ß = 34.720) and Peru (ß = 59.412), which indicates a lower increase in the distribution.

**Figure 1:**
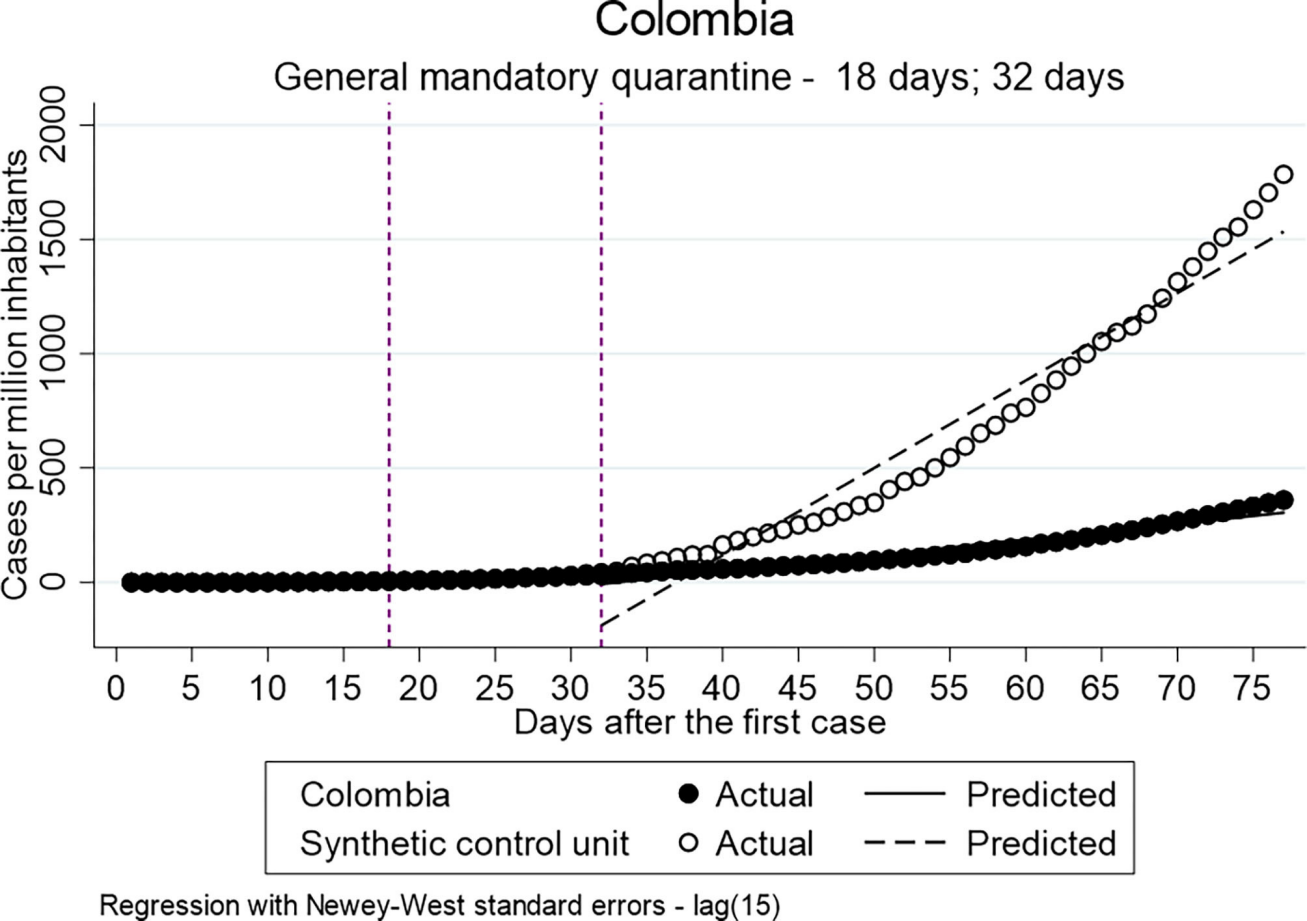
Results of ITSA general mandatory quarantine-Colombia.

**Figure 2:**
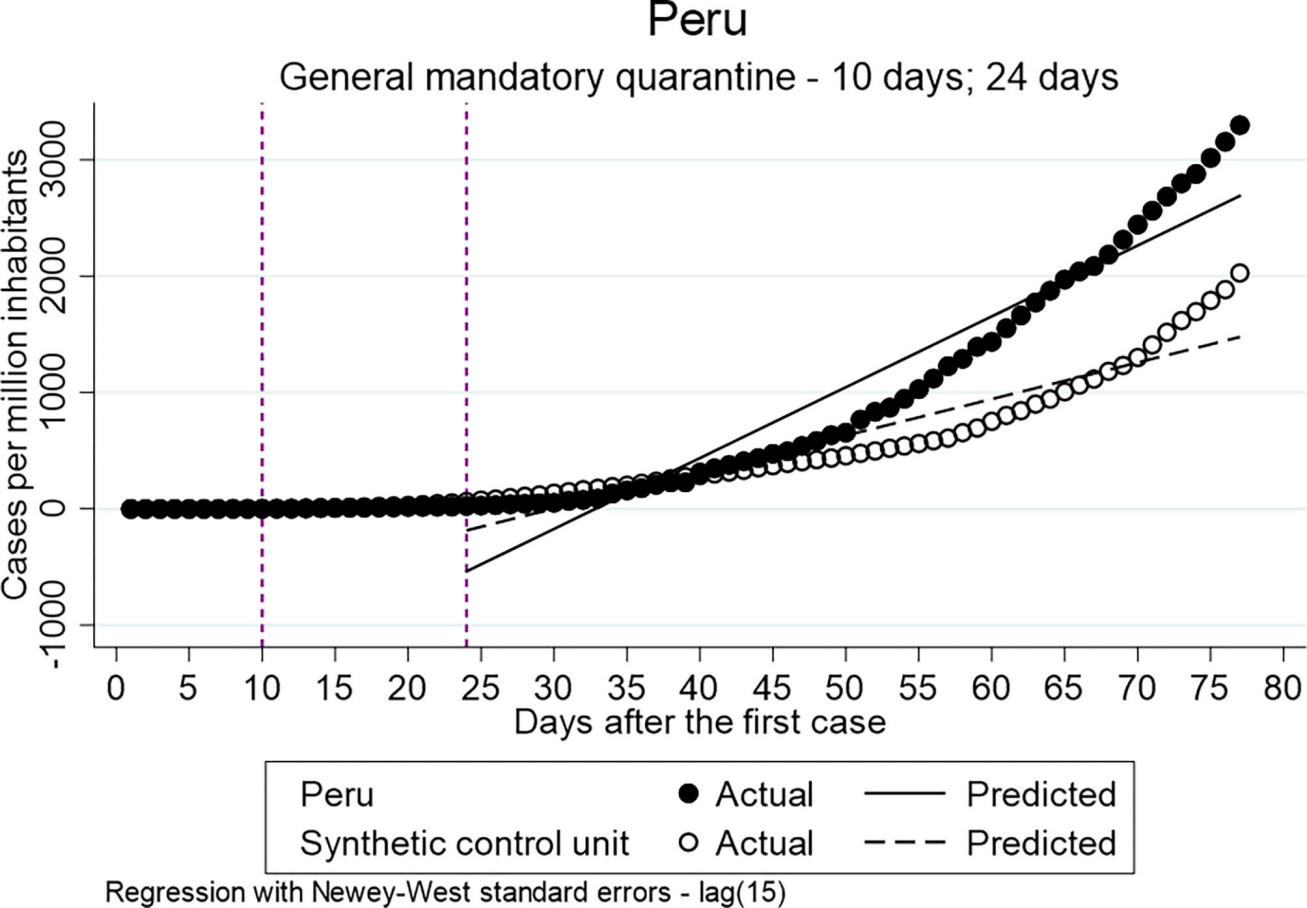
Results of ITSA general mandatory quarantine-Peru.

**Figure 3:**
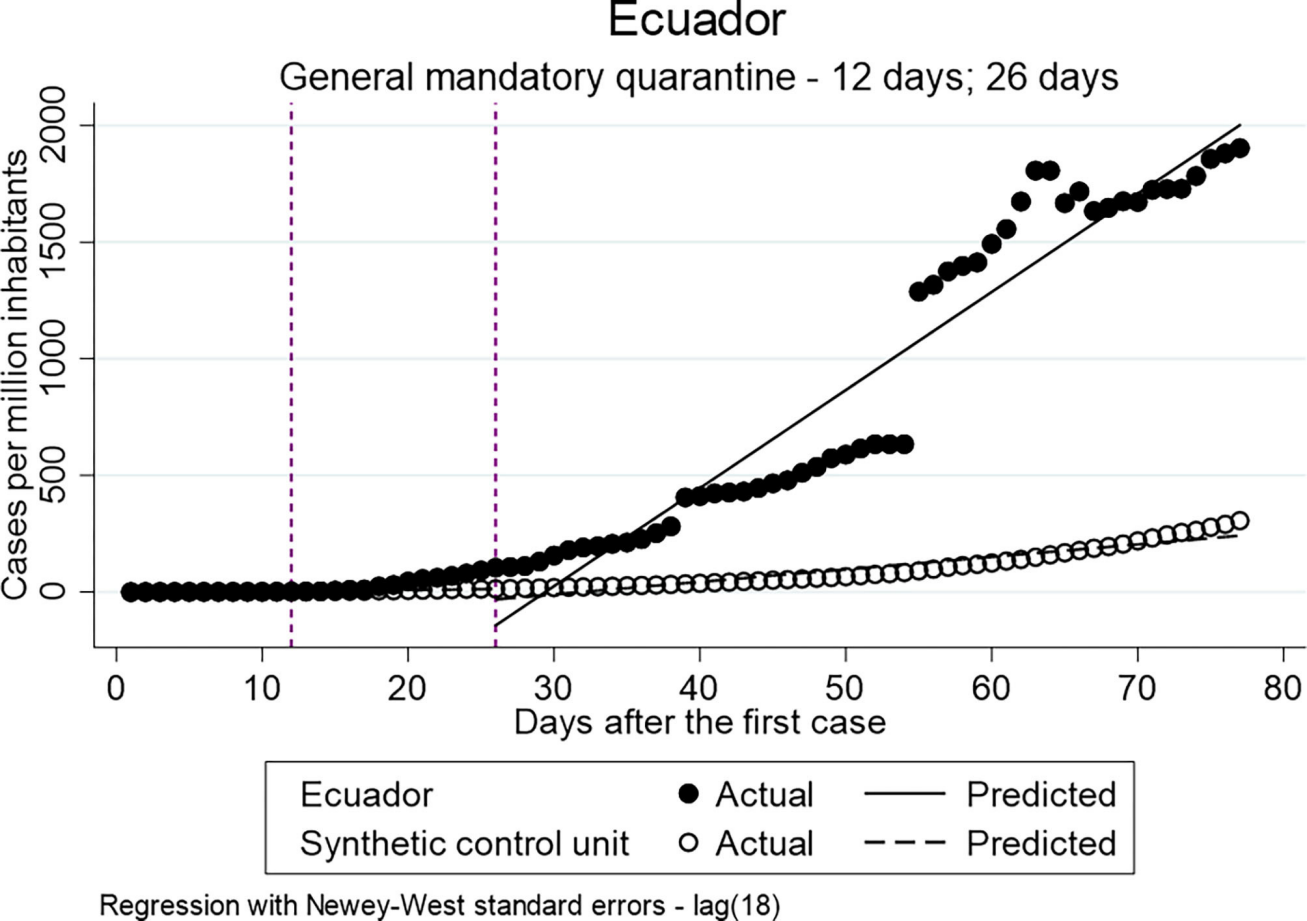
Results of ITSA general mandatory quarantine-Ecuador.

Figure 1 shows the distribution of the confirmed cases per million inhabitants vs. the counterfactual in Colombia. It indicates that the implementation of the general mandatory quarantine decreased the number of cases. Figures 2, 3 show a higher distribution of confirmed cases in Peru and Ecuador compared with the counterfactual. The multiple group ITSA in Table 7, Model 4B identifies a significant positive coefficient of the slope difference between pre-and post-Intervention periods for the treatment group at the first interruption time point (ß = 1.548) and 14 days after the implementation of the policy for Colombia (ß = 5.106). In other words, the analysis identifies a significant effect of the mandatory general quarantine in Colombia against the counterfactual.

**Table 7:** Results multiple ITSA–General Mandatory Quarantine.

	**Colombia**				**Ecuador**				**Peru**			
	**Implementation of**		**+** **14 days**		**Implementation of**		**+** **14 days**		**Implementation of**		**+** **14 days**	
	**the Intervention**		**delay**		**the Intervention**		**delay**		**the Intervention**		**delay**	
	**(Model 4a)**		**(Mode 4b)**		**(Model 5a)**		**(Model 5b)**		**(Model 6A)**		**(Model 6b)**	
	**β-Coefficient**	**Std. error**	**β-Coefficient**	**Std. error**	**β-Coefficient**	**Std. error**	**β-Coefficient**	**Std. error**	**β-Coefficient**	**Std. error**	**β-Coefficient**	**Std. error**
Pre-intervention intercept	−0.797	0.420	−0.797	0.426	−0.034**	0.012	−0.034**	0.112	−0.099	0.053	−0.099	0.054
Pre-intervention slope	0.257***	0.052	0.258***	0.052	0.128***	0.002	0.128***	0.002	0.146***	0.129	0.146***	0.013
Intercept differences between treatment and control pre-Intervention	−0.145	0.596	−0.145	0.604	0.236***	0.027	0.236***	0.027	−0.040	0.077	−0.040	0.078
Slope differences between treatment and control pre-Intervention	0.014	0.073	0.014	0.074	−0.032***	0.005	−0.032***	0.005	0.015	0.019	0.015	0.019
Immediately post (day of implementation)	−92.218	0.073	−0.278	0.836	−51.409*	24.367	−0.523	0.294	−314.366*	139.847	−6.348*	2.524
Difference between pre- and post-Intervention slopes (day of implementation)—control group	8.698***	1.516	2.152***	0.152	3.995***	0.730	0.732***	0.043	24.640***	4.453	3.614***	2.524
Difference between pre- and post-Intervention intercept (day of implementation)—treatment group	43.729	49.160	1.041	1.100	−322.660*	151.718	−13.419**	4.010	−367.046	332.908	5.916*	2.540*
Difference between pre- and post-Intervention Slope (day of implementation)—treatment group	−3.454*	1.705	−0.604**	0.199	30.136***	3.686	6.513***	0.547	21.087*	9.375	−2.292***	0.381
Immediately post (day of implementation) 14 days			−77.039*	38.157			−44.668*	20.798			−232.530	125.683
Difference between pre- and post-Intervention slopes (day of implementation)—control group 14 days			9.288***	1.652			4.497	0.767			27.608***	5.066
Difference between pre- and post-Intervention intercept (day of implementation)—treatment group 14 days			40.114	42.843			−188.721	100.574			−326.221	263.356
Difference between pre- and post-Intervention Slope (day of implementation)—treatment group 14 days			−4.181*	1.856			30.223***	3.296			31.804**	9.145

Significance levels: *p < 0.05, **p < 0.01, ***p < 0.001.

For both, Ecuador (Model 5a/b) and Peru (Model 6a/b), the estimates indicate higher values of confirmed cases per million inhabitants for the treatment group compared to the control group. The implementation of the mandatory general quarantine is hypothesized to have a reducing effect on the number of infected and death, therefore, the results of the multiple ITSA for Ecuador and Peru will not be discussed further. The hypothesis cannot be accepted.

To investigate whether the obligation to wear face masks in public transport and/or closed public spaces has a decreasing effect on the distribution of the confirmed cases per million inhabitants when single and multiple ITSA for Colombia and Chile were employed. The policy was implemented by Chile and Colombia 43 and 28 days after the first confirmed case, respectively.

Table 8 shows the results of the single ITSA. Statistically significant differences between the pre- and post-intervention periods can be identified by the analysis for both countries. However, similar to the previous models in Table 7, the coefficients indicate an increase in the confirmed cases per million inhabitants in the post-intervention period, and, therefore, it is indicated that the implementation did not have a decreasing effect on the distribution.

**Table 8:** Results single ITSA–Mask Obligation.

	**Chile**				**Colombia**			
	**Implementation of**		**+** **14 days**		**Implementation of**		**+** **14 days**	
	**the Intervention**		**delay**		**the Intervention**		**delay**	
	**(Model 7a)**		**(Model 7b)**		**(Model 8a)**		**(Model 8b)**	
	**β-Coefficient**	**Std. error**	**β-Coefficient**	**Std. error**	**β-Coefficient**	**Std. error**	**β-Coefficient**	**Std. error**
Pre-intervention intercept	−90.088*	37.745	−90.088*	38.272	−4.024*	1.706	−4.024*	1.730
Pre-intervention slope	10.113***	1.619	10.113***	1.641	0.720***	0.125	0.720***	0.127
Immediately post (day of implementation)	−186.244**	111.460	97.284*	39.287	−35.116	18.991	5.702**	1.952
Difference between pre- and post-Intervention slopes (day of implementation)	48.785***	7.837	13.840***	1.770	5.758***	0.804	2.479***	0.137
Immediately post (14 days delay)			−114.530*	52.841			−36.609*	13.975
Difference between pre- and post-Intervention slopes (14 days delay)			63.210***	5.239			5.228***	0.727

Significance levels: *p < 0.05, **p < 0.01, ***p < 0.001.

**Figure 4:**
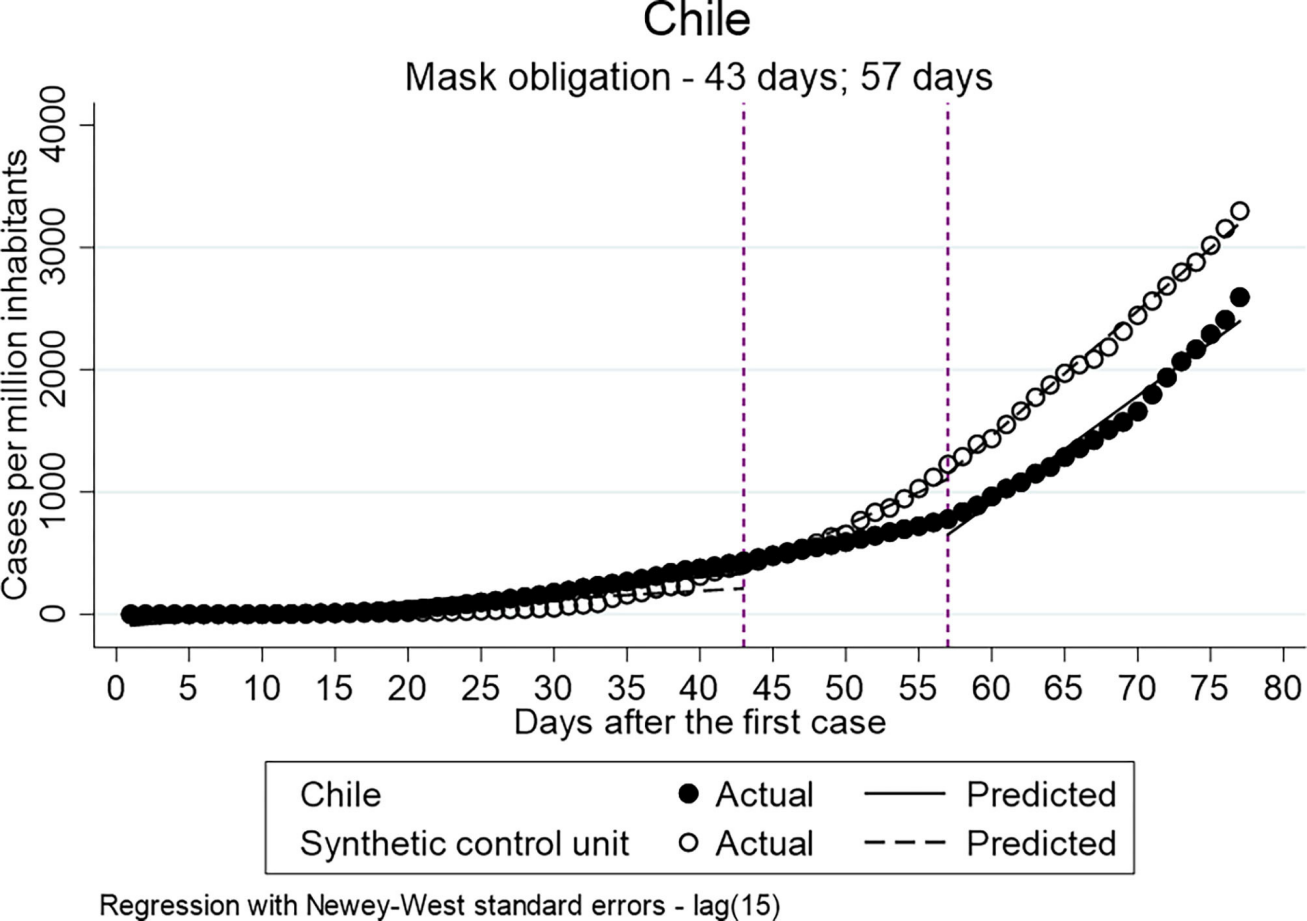
Results of ITSA mask obligation-Chile.

**Figure 5:**
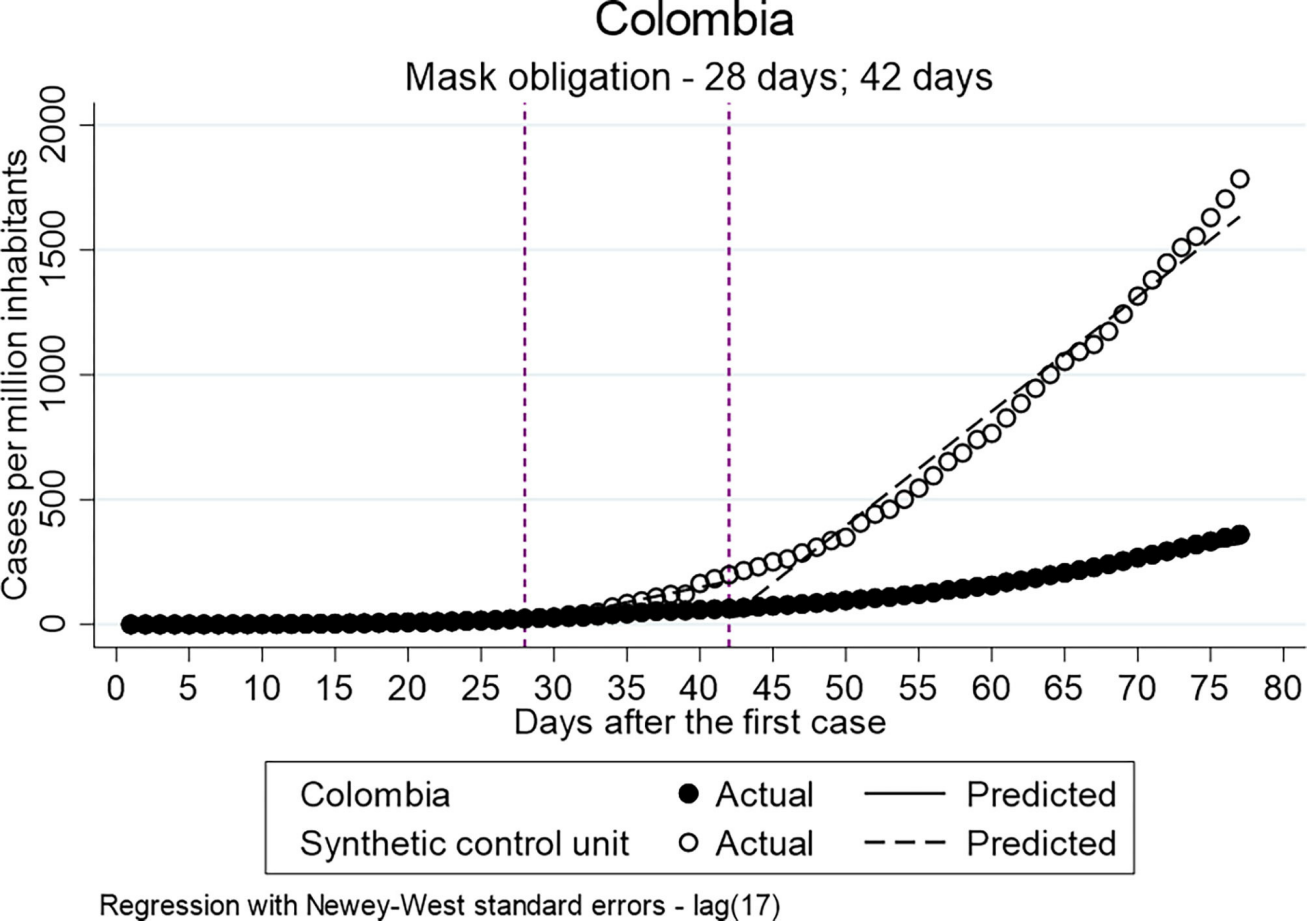
Results of ITSA mask obligation-Colombia.

The multiple ITSA using synthetic control units are displayed in Table 9. In Figures 4, 5 the results are visualized. The results of the model, including the delay of 14 days after the implementation, identify for both countries a significant decreasing effect of the policy on the distribution of confirmed cases per million inhabitants. The Intercept of the treatment group in Colombia indicates higher values for Colombia in comparison to the synthetic control units at both time points (ß = 16.854; ß = 108.233) even though the only the coefficient of the immediately post-intervention is statistically significant. The slopes between the pre- and post-Intervention period of the treatment group indicate significantly less confirmed cases compared to the control unit (ß = −8.863; ß = −28.598). In the case of Chile, only the coefficient of the difference between the pre- and post-Intervention period in the period immediately after the implementation is statistically significant and negative (ß = −33.717). The coefficient of the 14-day delay indicates a higher number of confirmed cases in the slope between pre-and post-intervention period for the treatment group compared to the control group, which is, however, not statistically significant (ß = 15.045). In summary, the results of the multiple ITSA for Chile and Colombia generally indicate that the introduction of compulsory masks has reduced the spread of the virus.

**Table 9:** Results multiple ITSA–Mask Obligation.

	**Chile**				**Colombia**			
	**Implementation of**		**+** **14 days**		**Implementation of**		**+** **14 days**	
	**the Intervention**		**delay**		**the Intervention**		**delay**	
	**(Model 9a)**		**(Model 9b)**		**the Intervention (Model 10a)**		**(Model 10b)**	
	**β-Coefficient**	**Std. error**	**β-Coefficient**	**Std. error**	**β-Coefficient**	**Std. error**	**β-Coefficient**	**Std. error**
Pre-intervention intercept	−69.970	37.753	−69.970	38.280	−3.716*	1.603	−3.716*	1.626
Pre-intervention slope	6.703**	1.909	6.703**	1.936	0.705***	0.115	0.705***	0.115
Intercept differences between treatment and control pre-Intervention	−20.118	55.024	−20.118	55.794	−0.309	2.367	−0.309	2.401
Slope differences between treatment and control pre-Intervention	3.410	2.538	3.410	2.574	0.015	0.168	0.015	0.171
Immediately post (day of implementation)	−93.226	74.074	135.830**	47.152	−263.564*	109.830	−11.163*	5.613
Difference between pre- and post-Intervention slopes (day of implementation)—control group	79.538***	5.948	47.556***	4.592	34.784***	3.936	11.342***	0.927
Difference between pre- and post-Intervention intercept (day of implementation)—treatment group	−93.018	136.201	−38.546	62.604	228.449*	111.521	16.864**	5.980
Difference between pre- and post-Intervention Slope (day of implementation)—treatment group	−30.753**	9.876	−33.717***	4.965	−29.026***	4.022	−8.863***	0.937
Immediately post (day of implementation) 14 days			42.725	28.994			−144.841*	55.686
Difference between pre- and post-Intervention slopes (day of implementation)—control group 14 days			48.168***	5.057			33.826***	3.295
Difference between pre- and post-Intervention intercept (day of implementation)—treatment group 14 days			−157.254*	66.735			108.233	57.528
Difference between pre- and post-Intervention Slope (day of implementation)—treatment group 14 days			15.045	7.829			−28.598***	3.374

Significance levels: *p < 0.05, **p < 0.01, ***p < 0.001.

## Discussion

The objective of the study was to show whether the proportion of deaths among COVID-19 cases and the efficiency of NPIs are influenced by macro indicators of the countries under study. Firstly, this study shows no clear influence of the macro indicator poverty headcount ratio at national poverty lines and urban population on the percentage of death within the confirmed cases. The same is true for the resources of the healthcare system and the access to those measured by OOP spending. However, Ecuador and Mexico report the highest percentage of death among the confirmed cases, and they report the highest share of OOP spending. The data indicates that higher OOP spending takes an impact on the percentage of death among the confirmed cases. Among the countries with a lower share of OOP spending, no trend is visible. Further research should therefore address the question whether OOP spending has an impact on the percentage of death among.

Secondly, the most important finding of this study concerns the effect of the implemented policies on the distribution of the confirmed cases. The first analysis showed a positive impact of the implementation of a mandatory general quarantine on the distribution of confirmed cases for Colombia but not for Ecuador and Peru. Peru and Ecuador share similar patterns in the OOP share of health spending, which is higher compared to Colombia. The percentage of poverty headcount ratio and national poverty lines is higher in Colombia compared to Ecuador and Peru. Health system resources are similar in all three countries. Even though the poverty in Colombia is higher, the access to drinking water and sanitation in Colombia is better compared to Peru and Ecuador (8). The need for access to sanitation and drinking water as basic human needs determine the possibility of keeping the quarantine, and, for this reason, it is conclusive that the inhabitants of Peru and Ecuador had less opportunity to carry out the quarantine compared to Colombia. In addition, factors such as informal employment increasing the need to leave to house in order to provide for living could play a role, which cannot be sufficiently verified due to lack of data [cf. (44)]. It must be considered that only countries that have implemented the policy during the time of observation can be considered in the discussion.

Finally, the analyses have shown that the introduction of mask obligation in Colombia and Chile has had a positive effect on the reduction of COVID-19 cases. In this sense, the analyses show that the effect of obligation to wear a mask is less influenced by external factors such as poverty compared to general quarantine. However, the mask obligation was only implemented by two out of six countries under observation. The result therefore only accounts for Chile and Colombia but not for the other countries.

The results indicate that the effect of the implemented policies depends on various factors and the implementation of a policy is not a guarantee of a flattened curve. These results go in line with those of previous studies, which showed that the efficiency of lockdown measures is influenced by various macro indicators such as population density (26).

Several limitations must be borne in mind when interpreting the findings of this study. Firstly, it must be considered that only reported and confirmed cases can be included in the analysis. This paper only refers to reported cases of COVID-19 diseases published by the respective countries. In this sense, the number of unreported cases, which is estimated differently depending on the reproductive value, cannot be included (45). The possibility of a bias due to a high number of unreported cases exists, depending on the testing frequency of the countries. As data on testing performed are not sufficiently available for the countries treated, it was not be possible to control for it [cf. (29)]. In addition, only policies from the country level were treated. Countries that have mainly implemented policies at the state level, as it is the case in Mexico, were treated as countries with no/fewer implemented policies. This approach was chosen to manage complexity, which also leads to a possible bias. Furthermore, not all countries publish data on health insurance coverage, which is why the share of OOP was chosen to include the health system [cf. (8)]. Moreover, additional resources of the healthcare system could not be included because in this case, too, there was no consistent transparent reporting by the countries at the time of the research. Future studies should therefore include (as much as possible) the additional resources and tests done by the states in order to control for those biases.

Healthcare system resources and OOP spending could only be included in the analysis to a certain extent. Since the focus was on the impact of the introduction of quarantine and the introduction of the obligation to wear a mask, only those countries that have introduced it could be compared. Countries that did not introduce the mask obligation were generally neglected in the analyses and played an important role in the formation of the synthetic control unit. In future studies, however, all countries should be analyzed, possibly including more measurement dates. The Model Fit must also be considered. The analyses show high standard errors for some coefficients, which indicate a bad model fit. Nevertheless, the standard errors of the coefficients relevant for the analysis are not too high.

The work provides above all an explorative overview in a field that is new and largely untreated. Previous analyses have mainly referred to industrialized countries but not to developing countries. Future research must therefore further address whether and how policies that have been effective in industrialized countries can make an impact in developing countries with different demographic characteristics and challenges.

## Data Availability Statement

The original contributions presented in the study are included in the article, further inquiries can be directed to the corresponding author.

## Author Contributions

AP contributed to the design and implementation of the research, to the analysis of the results and to the writing of the manuscript.

## Conflict of Interest

The author declares that the research was conducted in the absence of any commercial or financial relationships that could be construed as a potential conflict of interest.

## Footnotes

^1^Interlinked health problems.

^2^Direct payments made by individuals.
